# Complement C1QC as a potential prognostic marker and therapeutic target in colon carcinoma based on single-cell RNA sequencing and immunohistochemical analysis

**DOI:** 10.17305/bjbms.2022.7309

**Published:** 2022-06-23

**Authors:** Huiming Deng, Yan Chen, Yong Liu, Li Liu, Ronghua Xu

**Affiliations:** 1 Department of Gastrointestinal Surgery, Huazhong University of Science and Technology Union Shenzhen Hospital, Shenzhen, China; 2 Molecular Pharmacology Research Center, School of Pharmaceutical Science, Wenzhou Medical University, Wenzhou, China; 3 Department of Ultrasound, Hainan General Hospital, Hainan Affiliated Hospital of Hainan Medical University, Haikou, China

**Keywords:** C1QC, colon carcinoma, prognostic marker, single-cell RNA sequencing

## Abstract

Immune cell infiltration plays an essential role in the occurrence and development of colon cancer. However, the main tumor-associated immune cell infiltration and its gene regulation in colon cancer still need to be further clarified to provide a new perspective for diagnosing and treating this disease. For this study, single-cell RNA sequencing (scRNA-seq) expression profiles and TCGA colon cancer data sets were first acquired from the GEO database. Then, Seurat, Monocle, LIMMA, Clusterprofile, GSVA, and GSEABase algorithms were used to systematically examine the data. Potential target drugs corresponding to target genes were analyzed in the Drugbank database and detected by molecular docking. Immunohistochemistry was used to assess the level of C1QC expression in the tissue microarray. Single cell analysis suggested that neutrophil activation might be the critical regulatory pathway in colon cancer and that macrophages were the main cell population involved. Subsequent functional enrichment analysis on differential genes in macrophages suggested that C1QC may be a critical regulatory factor in the occurrence and progression of colon cancer and was closely related to the survival of patients. According to the drug target prediction, palivizumab is a targeted drug for C1QC, and molecular docking demonstrated that palivizumab binds to C1QC. In addition, tissue-microarray-based immunohistochemical analysis showed that C1QC was highly expressed in colon cancer tissue, and the prognosis of colon cancer patients with high C1QC expression was worse, closely related to age, lymphatic metastasis, and the TNM stage (Tumor, Nodes, and Metastases). Our findings suggest that C1QC may regulate the macrophages in colon cancer immune infiltration, which is expected to be a potential immunotherapy target for colon cancer, and beneficial for the diagnosis and prognosis of colon cancer patients.

## INTRODUCTION

With more than 1.85 million new cases and 850,000 fatalities per year [1,2], colorectal cancer has emerged as one of the fastest-growing cancers as well as the third leading cause of death globally, following the diet changes and aging of the population. In recent years, surgery, radiation and chemotherapy, targeted therapy, immunotherapy, and other treatment techniques have increased the overall survival (OS) of patients with advanced colon cancer. For patients with colon cancer, survival has been impacted by early diagnosis, post-operative recurrence, chemotherapy drug resistance, and severe adverse reactions [3,4]. Therefore, it is crucial to advance understanding of the pathogenesis of colon cancer and search for potential biomarkers to further improve the diagnosis and survival rates of patients with colon cancer.

Studies have shown that immune cells in the tumor microenvironment participate in the biological development of colon cancer cells in a variety of ways, including tumor angiogenesis, metastasis, and immune escape. These roles have a significant impact on the pathological evaluation, treatment decision, and prognosis of colon cancer patients [5-7]. Analyzing the immune cell subsets regulating the functions of colon cancer cells with significant differences will be beneficial for the diagnosis and proper treatment of colon cancer. As a result of advances in biotechnology, researchers have developed the cutting-edge single-cell-based transcriptome sequencing approach (scRNA-seq) for analyzing the tumor microenvironment and clearly distinguishing cell diversity and heterogeneity in the tumor [8].

In recent years, scRNA-seq has revealed the complexity of tumor-infiltrating cells in different cancer types, including tumor-associated macrophages [9-11]. Macrophages are heterogeneous cell types that contribute to the development and progression of cancer by generating vascular growth factors, promoting extracellular matrix remodeling and inhibiting immunity [12]. Clinicians have utilized immunotherapy to inhibit tumor-associated macrophages, including disruption of macrophage expansion and differentiation by blocking the interaction between the colony-stimulating factor 1 receptor and its ligand colony-stimulating factor 1 and interleukin-34 [13-15]. Moreover, monotherapy was found to only have a limited impact on cancer due to compensatory inhibition of body mechanisms [16,17]. Therefore, searching actively for genes that regulate macrophages involved in the development of colon cancer is crucial for immunotherapy.

The present study analyzed changes in cell populations in single-cell data of colon cancer and paracancer tissue and identified five cell populations. Using functional enrichment analysis, macrophage populations that significantly correlated with neutrophil activity were obtained. We also screened out the differentially expressed gene C1QC, which regulates the infiltration of macrophages in colon cancer and affects the prognosis of colon cancer patients. C1QC was confirmed to be highly expressed in colon cancer tissue and the prognosis of colon cancer patients with high expression of C1QC is worse. Palivizumab was identified as a possible target drug for C1QC by drug target predictions and a molecular docking assay. Our study revealed the specific immune infiltrating subsets of macrophages in colon cancer, as well as the related target genes and targeted drugs that regulate macrophages involved in colon cancer development. The findings have provided new methods for colon cancer diagnosis and treatment.

## MATERIALS AND METHODS

### scRNA sequencing analysis

GSE163974 expression profile data by high-throughput scRNA-seq using 10× Genomics including barcodes, genes, and matrix files were downloaded from the GEO database. The samples included three normal tissues (GSM4994385) and three cancer tissues (GSM4994386). The data analysis was as follows: (1) Expression spectrum acquisition: A matrix package was used to read barcodes, genes, and matrix files, and each single cell cycle was acquired by single cell experiment, scran, and a scatter function. The cell cycle was defined as the G1 phase: G1 score > 0.5 and G2/M score < 0.5; G2/M phase: G2/M score > 0.5 and G1 score < 0.5; S phase: G1 score < 0.5 and G2/M score < 0.5; unknown: G1 score > 0.5 and G2/M score > 0.5. (2) Data quality control: The Seurat function was used to filter data, and parameters were set as min. cells = 3 and min. features = 200, the proportion of mitochondrial genes < 0.05, and the number of cell genes ranged from 200 to 20,000. (3) Unsupervised clustering was used to select appropriate components to construct the cell cluster: (a) Data normalization was carried out using the global-scaling method; (b) the candidate genes with high expression variation were screened based on an average value algorithm; (c) PCA analysis was used to observe the batch effect between samples; (d) the seurat random sampling method was used to construct a background distribution of correlation values between characteristic genes and principal components, and the JackStraw algorithm was employed to screen suitable principal components for subsequent cell subgroup analysis; (e) the expression profile of each cell was converted into a highly correlated cell population using the KNN algorithm to complete cell cluster identification, resolution = 0.6; and (f) the genes with certain differential expression folds and detectable in most cells in each cluster were determined using a log-scale as subsequent markers, and the criteria were: min.pct = 0.25, logfc.threshold = 0.25; (3) Cell subsets definition: Cell subsets were reannotated by SingleR and scCATCH.

### Pseudotime analysis

The Monocle algorithm was used to analyze the developmental trajectories of genes with large changes. Genes with high expression variability in developmental trajectories were screened for subsequent analysis. The criteria were status = ok, family = tobit, *p* < 0.05, and order = true.

### Functional enrichment analysis of pseudotime regulatory genes

DEGs were obtained by pseudotime analysis and the cluster profile package was used for the GO analysis. The differential pathway was corrected by the BH method and the adjusted *p* < 0.05.

### Pathway enrichment and GSVA analysis

After the critical pathway, most related to the pseudotime-difference genes was obtained, the gene set of a pathway was downloaded from the MsigDB database (https://www.gsea-msigdb.org/gsea/msigdb/), and a standardized score was obtained for each cell using GSVA and GSEABase algorithms.

### TCGA analysis

RNA-seq Recompute TPM sample data of GDC TCGA-HNCC, clinical characteristics, and survival follow-up data were downloaded from the XENA database (https://xenabrowser.net/datapages/). DESeq algorithm was used to normalize the expression profile and filter low expression genes. The selection criteria for differential genes were a log2 fold change ≥1.0, and a *p*-value after the Benjamini-Hochberg procedure < 0.05. In addition, cluster profile packages were used for GSEA analysis of differential genes, and differential pathways were corrected by the BH method, with an adjusted *p* < 0.05.

### Hub genes identification and functional enrichment analysis

The development-related differential genes obtained by scRNA pseudotime analysis were divided into different modules according to different developmental states. Functional enrichment analysis was performed for each gene in different modules in the Toppgene database (https://toppgene.cchmc.org/), with screening criteria of FDR adjusted to *p* < 0.05.

### Hub genes screening and survival analysis

After obtaining the cell population with the most significant activation of the critical pathway through GSVA analysis, the genes with the most remarkable differences and the most significant *p*-value in the cell population were selected as candidate genes. In addition, the expression values of hub genes were used to analyze OS by the survival and survminer algorithm in each TCGA patient.

### Functional enrichment analysis of hub genes and prediction of drug targets

After obtaining target genes, functional enrichment analysis was conducted in the Toppgene database (https://toppgene.cchmc.org/). Potential target drugs corresponding to target genes were analyzed in the Drugbank database (https://go.drugbank.com/), and the screening criterion was *p* < 0.05 after FDR correction.

### Tissue microarrays and IHC assays

Colon cancer tissue microarray (TMA, HColA180Su18-XT17-025) was obtained from Shanghai Outdo Biotech, including 93 cases of colon cancer tissue and 87 cases of non-cancer tissue, with complete case and follow-up information. IHC assays and IHC scores were performed with a previously described protocol [18]. Briefly, the colon cancer tissue microarray was dewaxed and rehydrated using gradient alcohol after being baked in an oven for 1 h at 63°C. The endogenous peroxidase was blocked and inactivated by 3% H_2_O_2_. The tissue microarray was subject to antigen thermal repair with citric acid and was incubated overnight in a refrigerator at 4°C with C1QC primary antibody (Abcam, MA, US) and continued for 15 min at 37°C with secondary antibody (Zhong ShanGolden Bridge Biological Technology Co., Ltd, Beijing, China). The colon cancer tissue microarray was stained with DAB (Beyotime Biotechnology, Shanghai, China) and restained with hematoxylin (Sigma, CA, US). After dehydration and transparency, the samples were sealed and scanned.

The scoring system for IHC is as follows: The degree of immunostaining (A): 0 point for no obvious coloring, 1 point for mild, 2 points for moderate, or 3 points for strong; percentage of positive cells (B): <5%, 0 point; 6~25%, 1 point; 26~50%, 2 points; 51~75%, 3 points; and >75%, 4 points. The total score for each examined slide was the summed score of A+B. A high expression level was defined as the sum of scores ≥4 points, while a low expression level was considered as the sum of scores <4 point.

### Molecular docking

The protein structures of C1QC and palivizumab were downloaded from the PDB database and saved in the pdb format. Docking was performed by the zDock module from Discovery Studio 2019 to select the conformation with the highest score for subsequent analysis.

### Statistical analysis

Statistical analysis and chart preparation were performed by GraphPad Prism software 8.0 (GraphPad Software, Inc., San Diego, CA, USA). Student’s t-test was used for the comparison between the two groups. A Chi-squared test was used to analyze clinicopathological characteristics, and the Kaplan-Meier method and log-rank test for survival analysis. *p* < 0.05 was considered statistically significant.

## RESULTS

### Single-cell analysis results

**FIGURE 1:**
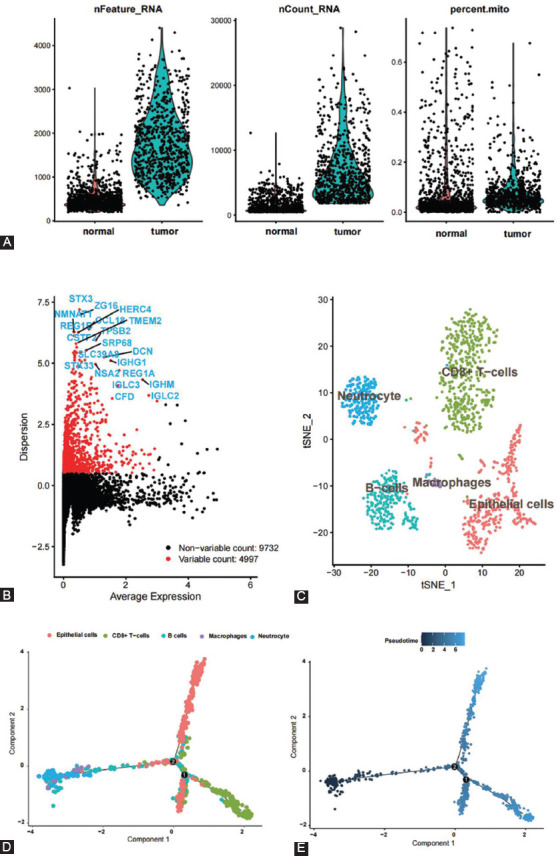
scRNA-seq clustering and pseudotime analysis in the GSE163974 dataset. (A) The sample quality control and proportion of mitochondria; (B) the highly variable genes are presented and the top 25 genes of each dataset are visualized; (C) UMAP map showing the scRNA cell clusters and annotation in each dataset; and (D and E) pseudotime analysis of the GSE163974 dataset.

After strict quality control, single-cell transcriptome data of 1211 cells were obtained, including 322 cells from cancer tissue and 889 cells from normal tissue (Figure 1A). After data standardization and correction, unsupervised clustering was performed on the combined dataset, and the cell types were annotated using known marker genes for specific cell types. 4,997 variable genes were identified, including STX3, ZG16, HERC4, NMNAT1, CCL18, and TMEM2 (Figure 1B). In addition, t-SNE and UMAP dimensionality reduction algorithms were used (two algorithms should correspond to two types of plots, when using two algorithms are redundant). The data set identified five cell populations, including CD8+ T-cells, neutrocytes, macrophages, B-cells, and epithelial cells. The distribution of cells is shown in two-dimensional space, with each point representing a cell (Figure 1C). We found a series of immune cells involved in gastric cancer by single-cell analysis.

### Pseudotime analysis and enrichment analysis

The expression profile data were projected into low-dimensional space to construct the differentiation trajectory among cells. Each dot represents a cell and cells in a similar cell state are grouped together. This study found that two branching points in the differentiation trajectory represent potential decision-making points in cellular biological processes (Figure 1D and E). Figure 1D is color-coded according to cell clustering and annotation information, while Figure 1E is color-coded according to the differentiation state.

**FIGURE 2:**
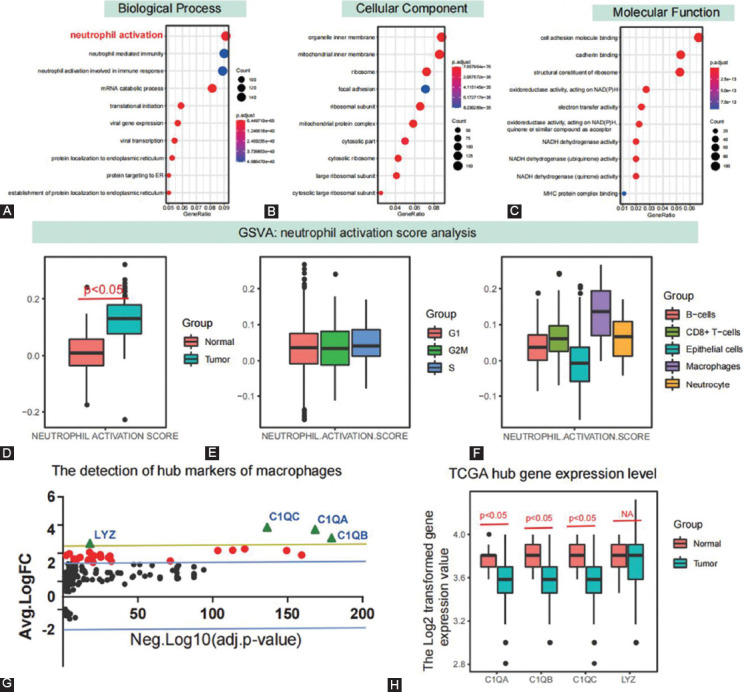
Functional enrichment analysis of pseudotime related differential expressed genes. (A) The GO terms analysis in response to pseudotime related differential expressed genes; (B) the tendency of GSVA calculated neutrophil activation scores involved in cancer samples, cell cycle, and different cell populations; (C) identification of the hub genes in response to development and progress of CRC involving macrophages; (D) the hub gene’s expression level in response to the TCGA-CRC dataset; (E and F) neutrophil activation score changes in different cell cycles and cell subsets in single cells; (G) distribution of differentially expressed genes in macrophages; and (H) differential expression changes of differential hub genes C1QA, C1QB, C1QC, and LYZ in TCGA tissue samples.

Developmental trajectory analysis was conducted based on the gene sets with great changes. Log standardization and DDRTree dimension reduction were carried out and B-H corrected differential regulatory genes were selected for subsequent functional enrichment analysis after pseudotime analysis. Figure 2A shows that the BP pathway is associated with cancer differentiation and progression, including neutrophil activation (p.adjust = 2.63 × 10-41; enriched genes = 158), neutrophil-mediated immunity (p.adjust = 4.99 × 10-40; enriched genes = 156), and neutrophil activation involved in immune response (p.adjust = 4.99 × 10-40, enriched genes = 154).

Moreover, organelle inner membrane (p.adjust = 2.72 × 10-43, enriched genes = 158), mitochondrial inner membrane (p.adjust = 2.71 × 10-46, enriched genes = 154), and ribosome (p.adjust = 1.11 × 10-62, enriched genes = 130) were significantly enriched in cellular components (Figure 2B). Cell adhesion molecule binding (p.adjust = 5.27 × 10-46, enriched genes = 90), cadherin binding (p.adjust = 4.34 × 10-21, enriched genes = 91), and structural constituent of ribosome (p.adjust = 1.34 × 10-19, enriched genes = 114) were mainly enriched in molecular function (Figure 2C). Pseudotime analysis indicated that neutrophil mediated immune pathway may play an essential role in the development and progression of gastric cancer.

### GSVA analysis of neutrophil activation and changes in various cell populations

Using the ssGSEA algorithm, we calculated neutrophil activation pathway scores for each cell. Differential analysis revealed that neutrophil activation GSVA scores were significantly different between cancer and normal tissue (*p* < 0.05; Figure 2D). We also found no significant differences in neutrophil activation and the cell cycle (*p* > 0.05; Figure 2E). Macrophages exhibited the most significant change in the neutrophil activation GSVA score among all cell populations (Figure 2F). Therefore, we selected macrophages as the critical regulatory cells. Based on the quantitative difference analysis of GSVA pathway, we further confirmed the important role of neutrophil mediated immunity pathway in gastric cancer, and the change of this pathway may be caused by macrophages.

### Identification of hub genes in the macrophage population

Differentially expressed genes in macrophages were selected and analyzed by expression differential folds and corrected *p*-values. Genes with large expression differences and the most significant *p*-value were selected as candidate regulatory genes, namely, C1q A-peptide (C1QB), C1q B-peptide (C1QB), C1QC, and LYZ (Figure 2G). There were significant differences in the expression of C1QA, C1QB, and C1QC in the TCGA colon cancer dataset (*p* < 0.05), but no significant differences in LYZ (Figure 2H). C1QA, C1QB, and C1QC may be the hub genes for macrophages regulating neutrophil activation.

### Pseudotime regulation gene enrichment analysis

**FIGURE 3:**
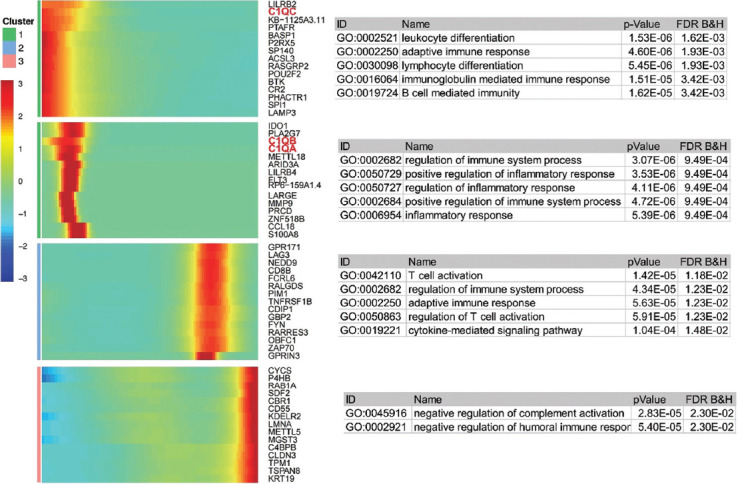
The pseudotime-related DEGs and functional enrichment for each cluster. Function enrichment analysis of pseudotime-related DEGs and biological processes.

Through pseudotime analysis, the most significant differential expression gene sets in different developmental states were obtained and enrichment analysis of each gene set was carried out. In the first stage of development, the main expressed genes were LILRB2, C1QC, and KB−1125A3.11, which were enriched in GO:0002521 leukocyte differentiation (*p* = 1.53 × 10-06; FDR = 1.62 × 10-03), GO:0002250 adaptive immune response (*p* = 4.60 × 10-06; FDR = 1.93 × 10-03), and GO:0030098 lymphocyte differentiation (*p* = 5.45 × 10-06; FDR = 1.93 × 10-03) pathways. In the second stage, the main expressed genes were IDO1, PLA2G7, C1QB, and C1QA, and enriched in GO:0002682 regulation of immune system process (P-value = 3.07× 10-06; FDR = 9.49 ×10-04), GO:0050729 positive regulation of the inflammatory response (*p* = 3.53 × 10-06; FDR = 9.49 × 10-04), and GO:0050727 regulation of the inflammatory response (*p* = 4.11 × 10-06; FDR = 9.49 × 10-04) pathways. GPR171, LAG3, and NEDD9 were the main expressed genes in the third stage, which were enriched in GO:0042110 T cell activation (*p* value = 1.42 × 10-05; FDR = 1.18× 10-02), GO:0002682 regulation of immune system process (*p* = 4.34 × 10-05; FDR = 1.23 × 10-02), and GO:0002250 adaptive immune response (*p* = 5.63 × 10-05; FDR = 1.23 × 10-02) pathways. The fourth stage included CYCS, P4HB, RAB1A, and GPR171, which were enriched in GO:0045916 negative regulation of complement activation (*p* = 2.83 × 10-05; FDR = 2.30 × 10-02) and GO:0002921 negative regulation of the humoral immune response (*p* = 5.40 × 10-05; FDR = 2.30 × 10-02) pathways (Figure 3). Pseudotime analysis showed that C1QC critically regulates macrophages.

### GSEA analysis of differential genes in TCGA colon cancer

**FIGURE 4:**
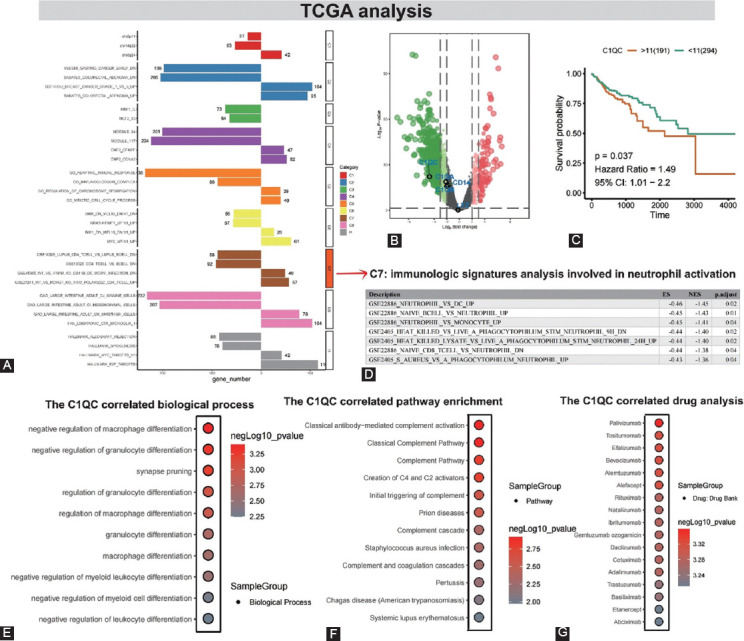
Identification of hub genes and their expression patterns in CRC cancer development in TCGA dataset. (A) The bar plot shows results of the GSEA analysis in CRC development; (B) the volcano map presents the CRC-related differential gene distribution; (C) the overall-survival analysis in response to C1QC based on the TCGA database. The survival curve is based on survival and survminer functions. The optimal cutoff values of multiple gene variables are calculated by an automatic cut-off algorithm, and the survival curve is drawn; (D) summary of GSEA analysis of TCGA differential genes: In C7, the immune signature analysis involved in neutrophil activation mainly enriched GSE22886 NEUTROPHIL VS DC UP, GSE22886 NAIVE BCELL VS NEUTROPHIL UP and GSE22886 NEUTROPHIL VS MONOCYTE UP core pathways. Further validation of neutrophil activation in the progression of colon cancer; and (E-G) the biological process, targeted drugs, and pathway enrichment analysis for C1QC.

The count dataset in GDC TCGA Colon Cancer (COAD) was downloaded from the UCSC Xena database (https://gdc.xenahubs.net), and 1685 differential genes by DEseq calculation were obtained (Figure 4A and B). GSEA analysis revealed that GSE22886_NEUTROPHIL_VS_DC_UP (ES = −0.46, NES = −1.45, p.adjust = 0.02), GSE22886_NAIVE_BCELL_VS_NEUTROPHIL_UP (ES = -0.45, NES = −1.43, p.adjust = 0.01) and GSE22886_NEUTROPHIL_VS_MONOCYTE_UP (ES = −0.45, NES = −1.41, p.adjust = 0.04) were significantly enriched in the C7 module (Figure 4D). Transcriptome analysis of TCGA gastric cancer tissues suggested that neutrophil activation plays a key role gastric cancer progression.

### C1QC survival analysis and functional enrichment analysis

**FIGURE 5:**
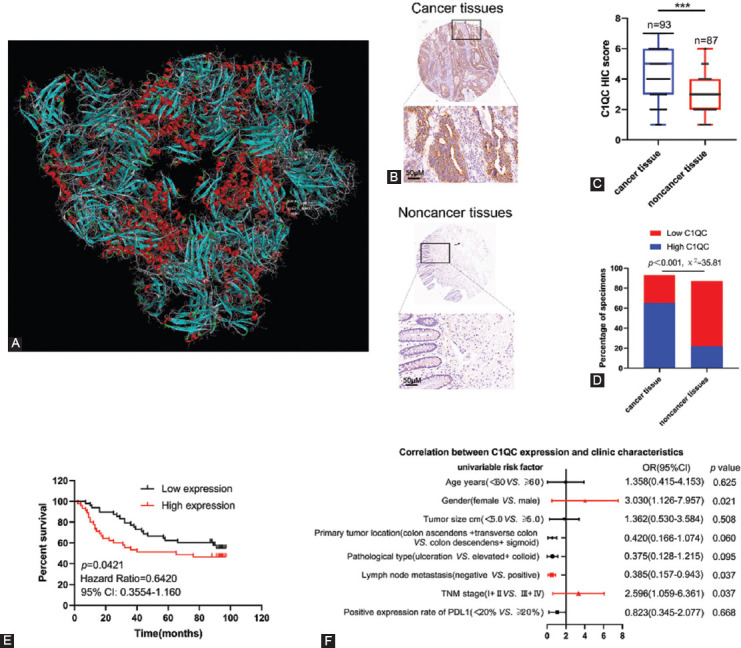
Expression levels of C1QC in colon cancer tissue and non-cancer tissue and its relationship with clinical characteristics. (A) Palivizumab docking with C1QC; the three-dimensional diagram shows the binding conformation of palivizumab (red) with C1QC (azure); (B) representative immunohistochemistry staining of C1QC expression in colon cancer tissue and non-cancer tissue; (C and D) statistical analysis of immunohistochemical scores of C1QC, and percentage of high expression and low expression of C1QC in colon cancer tissue and non-cancer tissue; (E) Kaplan-Meier survival analysis of C1QC expression in patients with colon cancer; and (F) correlations of C1QC expression levels in colon cancer tissue and clinicopathological features of colon cancer patients. The statistical significance was determined by the χ2-test. Data are expressed as the mean ± SD.

Survival analysis showed that only C1QC was strongly associated with poor long-term survival in colon cancer (Hazard Ratio = 1.49, 95% CI:1.01−2.2, *p* = 0.037) (Figures 4C and Supplementary Figure 1). According to the above results, we chose C1QC for further analysis. Association pathway analysis suggested negative regulation of macrophage differentiation (*p* = 4.96 × 10-04), negative regulation of granulocyte differentiation (*p* = 3.97 × 10-04), and synapse pruning (*p* = 5.96 × 10-04) were mainly enriched in C1QC (Figure 4E). In addition, classical antibody-mediated complement activation (*p* = 1.20 × 10-03), classical complement (*p* = 1.20 × 10-03), and complement (*p* = 1.60 × 10-03) pathways were similarly identified (Figure 4F). Drug target prediction suggested that palivizumab (*p* = 4.38 × 10-04) was most likely to interact with the C1QC protein structure (Figure 4G), which was also verified by molecular docking experiments. The binding score of palivizumab to C1QC protein was 21.44 (Figure 5A).

### C1QC was highly expressed in colon cancer tissue and correlated with its poor prognosis

To verify the high expression of C1QC in colon cancer tissue and its correlation with OS of colon cancer patients, we detected the expression of C1QC in 93 colon cancer tissue and 87 non-cancer tissue. IHC results showed that the expression score of C1QC in colon cancer tissues was higher than that in non-cancer tissue (Figure 5B and C). In addition, the positive expression rate of C1QC in colon cancer tissue (IHC score ≥ 4) was also higher than that in non-cancer tissue (Figure 5D). Kaplan-Meier survival analysis showed that high C1QC expression was significantly correlated with poor OS (Figure 5E). As shown in Figure 5F, Table 1, and Supplementary Figure 2, C1QC expression in colon cancer was positively correlated with gender, lymph node metastasis, and the TNM stage. No significant correlation was found between C1QC expression and clinicopathological features, such as age, tumor size, primary tumor location, pathological type, or the positive expression rate of PDL. The expression of C1QC was higher in the colon cancer tissue of males, I+II (TNM stage), and lymph node metastasis patients. C1QC expression is closely related to the long-term prognosis of gastric cancer patients.

**TABLE 1:**
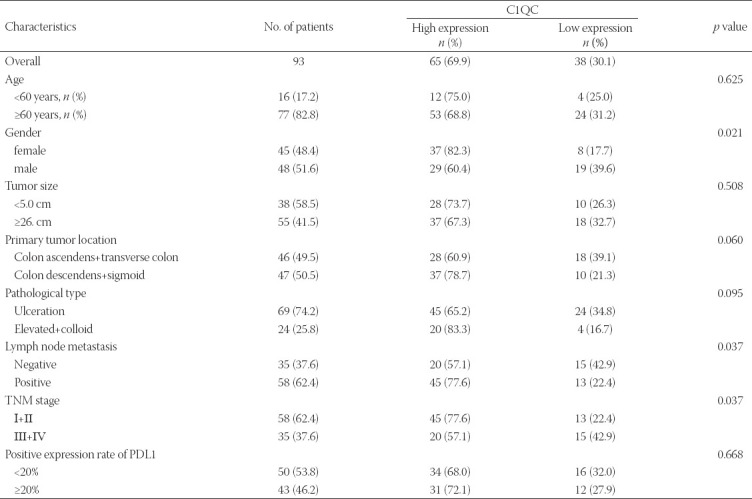
Correlation of the expression of C1QC in colon cancer with clinicopathologic features

## DISCUSSION

Immunotherapy has been further developed for different types of solid cancer, including colon cancer, after being successfully applied for melanoma. Patients with metastatic colon cancer can significantly prolong their survival with immunotherapy [19]. Identifying the biological functions of immune cell subsets and the related genes regulating their functional status is the key to cancer immunotherapy. Multiple scRNA-seq analysis studies showed differences in immune cell subsets across cancer types [9,20,21]. The differences in immunotherapy efficacy among different cancer are related to the diversity of infiltration of immune cell subsets. At present, the function and prognostic value of immune cell subsets are mainly analyzed by combining different biomarkers of immune cells with immunohistochemical spatial localization [22,23].

In the present study, 4997 variable genes were identified in colon cancer cells and paracancer tissue cells. CD8+ T-cells, neutrocytes, macrophages, B-cells, and epithelial cells were identified by t-SNE and the UMAP dimensional reduction algorithm. Then, the differentiation trajectories of these cell populations were constructed, and crucial decision points of biological process were revealed. After the neutrophil activation GSVA analysis, macrophages displayed the greatest change in the neutrophil activation GSVA score. Studies have shown that the characteristics of macrophages depend on the tumor tissue source. Compared with CD8+ T cells, neutrophils, B cells, and epithelial cells, macrophages in colon cancer tissue have a more substantial neutrophil activation effect, but this action was not significantly correlated with the cell cycle [9,10,21].

In line with earlier research, but in contrast to the M1 and M2 dichotomous polarization of macrophages and the different continuity of tumor-associated macrophages in breast and lung cancer [9,24], our results also demonstrated that there was no significant difference between neutrophil activation and the cell cycle in colon cancer cells. Macrophages may have a different functional status in various tumor types, which needs to be clarified in further studies. However, compared with normal colon tissue, macrophages in colon cancer tissue showed obvious immune response functions, suggesting that macrophages are the critical cells among the infiltrated immune cells in colon cancer.

Based on the above results, we analyzed the hub genes that regulate the infiltration of the macrophage population in colon cancer. Finally, we selected complement C1Q-related molecules C1QA, C1QB, C1QC, and LYZ as candidate genes. Among them, the expression of C1QA, C1QB, and C1QC showed significant differences in the TCGA colon cancer dataset, while LYZ exhibited no significant difference. Complement is a class of peptides with enzyme activity and self-regulation and the complement system regulates both innate and adaptive immune responses [25]. Complement C1q is produced by macrophages, immature dendritic cells, and mast cells. It is a subunit of complement C1 and regulates various immune cells. Under normal physiological conditions, the activation of complement C1q can effectively remove immune complexes, apoptotic cells and residual bodies, and maintain the immune response level in balance [26]. Studies have shown that complement C1q has a dual regulatory effect on macrophage activation and polarization [25] and promote liver metastasis of pancreatic ductal adenocarcinoma and progression of liver cancer [27,28]. In addition, tumor cells can hijack macrophages to produce complement C1q promoting tumor growth [29]. In terms of C1q-related molecules, C1QC+ tumor-associated macrophages can predict the prognosis, tumor stage, and immune cell infiltration of patients with cervical cancer, which helps treat cervical cancer [30]. The differential expressions of C1QA, C1QB and C1QC were found to be closely related to the survival prognosis, pathological features, and tumor microenvironment of osteosarcoma, which can help improve the clinical prognosis and immunotherapy [31]. The evidence suggests that C1q and its related molecules play an essential roles in macrophage activation and tumor development.

To explore the effects of C1QA, C1QB, and C1QC on macrophages in colon cancer, we conducted pseudotime analysis. The results demonstrated that among the candidate genes, C1QC was involved in white blood cells and lymphocyte differentiation and the adaptive immune response in the early stages, while C1QA and C1QB regulated immune system processes and inflammatory responses in the second stage. These two stages are also crucial in the biological roles of macrophages in the development of colon cancer. Therefore, it is speculated that the expression levels of C1QA, C1QB, and C1QC are closely related to the development of colon cancer. We also analyzed the relationship between C1QA, C1QB, C1QC and the survival of colon cancer patients, and the results showed that a high expression of C1QC in colon cancer patients led to a poor prognosis (*p* < 0.05). However, the apparent relationship between the high expression of C1QA and C1QB and the prognosis of colon cancer patients still needs to be further explored as statistical significance was not reached, which may be due to insufficient sample size. Future studies should expand the sample size to address this issue.

Since the current analysis was based on TCGA bulk transcriptome data, this inevitably led to some limitations of the findings. To further verify the result, we analyzed the gene characteristics of C1QC in 93 clinical specimens of colon cancer patients and 87 non-cancer tissues. IHC results showed that C1QC was highly expressed in colon cancer tissues, and the expression of C1QC in colon cancer was positively correlated with gender, lymph node metastasis, and the TNM stage, but there was no significant correlation with age, tumor size, primary tumor site, pathological type, or the PDL1-positive expression rate. Furthermore, the results illustrated the poor prognosis of colon cancer patients with high expression of C1QC. In addition, the drug target prediction and molecular docking experiments showed that palivizumab is the most likely prospective drug to target C1QC. The drug target docking analysis found that palivizumab can bind effectively to the C1QC protein.

## CONCLUSION

scRNA-seq analysis and validation of clinical samples showed that C1QC is a potential diagnostic biomarker and immunotherapy target for colon cancer patients. Clarifying the role of macrophage subsets characterized by the C1QC gene in the development of colon cancer, as well as the mechanisms of C1QC regulating immune infiltration of macrophage subsets will be essential. Further research is also required to determine the role of palivizumab as a C1QC targeted drug for colon cancer therapy. Although these issues remain to be addressed, our findings help identify new diagnostic and therapeutic approaches for colon cancer patients.
